# Effect of Group Cognitive Behavioral Therapy on Hardiness and Coping Strategies Among Infertile Women Receiving Assisted Reproductive Therapy

**Published:** 2012

**Authors:** Leili Mosalanejad, Anahita Khodabakshi Koolaee, Safie Jamali

**Affiliations:** 1 Assistant Professor, Jahrom University of Medical Sciences, Jahrom, Iran.; 2 Assistant professor, Rehabilitation and Welfare University, Tehran, Iran.; 3 Gynecology Department, Jahrom University of Medical Sciences, Jahrom , Iran.

**Keywords:** Assisted Reproductive Therapy, Cognitive Behavioral Therapy, Coping Strategy, Infertility

## Abstract

**Objective:** To determine the effect of group Cognitive Behavioral Therapy (CBT) on psychological hardiness and coping strategies of infertile women undergoing Assisted Reproductive Therapy (ART).

**Methods: **Two groups of infertile women who received ART consisted of experimental group (15 females) and control group (16 females) were compared. The participants in experimental group received 15 sessions of CBT group therapy. The control group received no psychological intervention. For gathering data Moss and Billing Coping Strategies Scales and AHVAZ Hardiness Scale for infertile women were used.

**Results:** There was a significant difference in hardiness between the two groups after intervention. Coping strategies did not differ significantly between the two groups before intervention, nor after intervention but there was significant difference regarding cognitive-oriented coping style in terms of scores of pre- and post-test in experimental group (P<0.05).

**Conclusion: **Group CBT developed psychological hardiness and cognitive-oriented coping style in experimental group.

## Introduction

Approximately 10-15% of couples in childbearing age experience infertility. Infertility is a stressful event that can give rise to psychological difficulties (1).Research has shown that psychological stress experienced by women with infertility is similar to that of women coping with illnesses like cancer, HIV, and chronic pain (2).A common theme in explaining the stressful impact of infertility is that it represents a life crisis and people may experience grief reactions similar to those seen in bereavement (3, 4).

In a review of the literature, it was found that infertility-related stress increased marital conflicts, decreased sexual satisfaction, sexual functioning, life quality, self-efficacy, intimacy and health (5).Fertility treatments are both a physical and emotional burden on women and their partners. Psychological factors such as depression, anxiety, and stress-induced changes in heart rate are predictive of a decreased probability of achieving a viable pregnancy. In fact, infertility is a complex crisis of life that is a psychological threat and an emotional pressure (5, 6).

A couple that is trying to conceive will undoubtedly experience feelings of frustration and disappointment if a pregnancy is not easily achieved. However, if the difficulties progress and the man and or woman are labeled as having fertility problems, then this may result in a severe insult to self-esteem, body image, and self-assessed masculinity or femininity(7).

Women in particular suffer from the psychological stress that can be caused by infertility; they are more anxious, depressed, and have a decreased self-esteem than their partners. The desire to counteract these emotional strains and to enhance the quality of life is increasing and accordingly requests for counseling services are on the rise(8).

Psychosocial treatments are known to not only prevent and lessen various mental problems such as anxiety, depression, and phobia, but also to play a positive role in physical health and a successful pregnancy (9).Psychological interventions have been found to be useful in alleviating depression in infertile couples before receiving infertility treatments (10).

Hardiness as a specific personality trait will impact individual’s resistance in collating with stress (11).Higher health stressors are associated with higher perception of illness impact (12).

Hardiness is a constellation of personality characteristics that serves as a resistance resource when encountering stressful situations. Three basic components comprise hardiness include challenge, which is the perception of change as normal and natural, as well as an opportunity for personal growth; commitment, which is a sense of purpose or meaningfulness in one’s life and a strong involvement in directing one’s life course; and control, which is the belief that one is capable of impacting one’s life circumstances (12, 13).

Evidence suggests that hardiness positively influences perceptions of stressful life events. In addition, hardiness has a beneficial relationship to self-ratings of physical health and physical symptoms, as well as to depression and anxiety and mental health (14, 15).

Coping style refers to an individual’s preferred behavioral and cognitive responses to stressful situations that remain stable across time and circumstances. For instance, approach-oriented coping styles have been associated with less global stress, and beneficially related to physical health indicators such as illness time loss, as well as indicators of mental health such as anxiety, depression, and psychological distress (16-19).

Although, many studies have examined hardiness as a protective factor against stress reaction, but no studies have yet examined the impact of group behavioral therapy on infertility hardiness. The aim of this study was to develop and assess the efficacy of group CBT on coping strategies and hardiness of known infertile women ART approach.

## Materials and Methods

The study population consisted of infertile women who presented to Ob/Gyn clinics of our university hospital for ART approach. Infertility was defined as at least 1 year of unprotected coitus without conception.

In this experimental study we used Ahvaz Hardiness inventory AHI and Billings and Moos coping questionnaire (20). All the questionnaires were confirmed and normalized for Iranian populations (21, 22). Billings and Moos coping questionnaire is a scale to measure coping strategies used by infertile women using a 4-point assessment scale (never to always) (23). Also, for gathering the data we used the AHI. This inventory contains 27 questionnaires. AHI comprises 4-point Likert scale (never to always)(20).

All infertile women who showed interest to participate at group CBT sessions were asked to participate in this study. The infertile subjects who agreed to participate had 18-35 years of age with no somatic or psychiatric problem and provided informed consent. The two groups were matched regarding age, socio-economic status (SES), education, and duration of infertility. Thirty-one consecutive infertile women were included in hormonal therapy or other ART approaches. They were randomly divided into two groups. Experimental group (16 subjects) received CBT but control group did not. In experimental group, 15 sessions of group CBT were held weekly in a period of four months. All sessions were directed by psychologist and the questionnaires were filled out by participants as a self-rating questionnaire. After sampling in the two groups, to adhere to cooperation of control group to complete the questionnaire forms, we used 1 educational session about new method of infertility treatment for this group after finishing the research.

CBT included the recognition of negative thinking to help the participants distinguish imagination from reality, cognitive reconstructing, thought blocking, spirituality, and the behavioral techniques included muscle relaxation exercises, imagination exercises, expressing feelings, and biofeedback. 

**Table 1:** Hardiness scorers in experimental and control groups before and after intervention

**CBT** ^†^ ** Intervention**	**Experimental**	**Control**	**State**	
	M±SD	M±SD	T	P
**Before**	46.82±16.04	43.44±2.59	2.11	0.42
**After**	52.40±02.36	40.72±2.33	0.021	0.001*

*p<0.05 is significant

^†^CBT=cognitive behavioral therapy

## Results

Baseline measure (hardiness, coping strategies and coping styles) of the experimental and control groups were not significantly different before intervention. Table 1 indicates that hardiness was not different significantly between the two groups before intervention. But there was a significant difference in hardiness between the two groups after group CBT. There was no significant difference within each group before and after intervention. Coping strategies did not differ significantly between the two groups before intervention, nor after intervention (Table 2).

**Table 2:** Differences in coping strategies between the two groups before and after intervention

**CBT Intervention**	**Coping** **Strategies**	**Experimental **	**Control**	**State**	
		M±SD	M±SD	T	P
**Before **	Problem oriented	19.05±5.37	17.72±6.27	0.59	0.50
	Affection oriented	10.76±1.30	09.66±3.06	7.32	0.18
**After **	Problem oriented	21.88±5.30	11.33±3.06	0.21	0.70
	Affection oriented	11.76±3.57	18.94±5.34	0.28	0.14

Moreover, Tables 3 indicates that coping strategies did not differ significantly before and after intervention within the experimental group, nor in the control group.

**Table 3:** Differences in coping strategies within groups before and after intervention

**Intervention** **Group**	**Coping** **Strategies**	**Before**	**After**	**State**	
		Mean(SD)	Mean (SD)	T	P
**Experimental **	Affection oriented	10.76±1.30	11.76±3.57	-1.25	0.22
	Problem oriented	19.05±5.37	21.88±1.54	-1.74	0.10
**Control**	Affection oriented	17.72±6.27	18.94±5.34	-0.83	0.417
	Problem oriented	09.66±3.06	11.33±3.06	-1.87	0.078

Regarding coping skills, it was revealed that after intervention mean scores of "attention to various methods to solve problem”, “positive experiences”, “praying” and “projection” in experimental group and “praying”, “intellectual decision making”, and “counseling” in control group were higher than other coping skills. 

**Table 4:** Differences in coping style between the two groups before and after intervention

**CBT Coping**	**Intervention**	**Experimental**	**Control**	**State**	
		M±SD	M±SD	T	P
Avoidant oriented	Before	08.47±1. 97	07. 61±0.60	1.10	0.27
	After	08.88±3.60	09.16±2.93	0.25	0.80
Cognitive oriented	Before	10.88±3.60	10.22±3.49	0.52	0.60
	After	13.05±4.29	11.27±3.5	1.34	0.19
Behavioral oriented	Before	10.47±2.42	09.55±3.43	0.90	0.36
	After	11.70±3.45	09.83±2.77	1.77	0.08

Also comparing coping skills before and after intervention showed that changes in "consulting with her husband and family to solve the problem" (1.7±0.89 vs. 2.1±0.96; p=0.04), "Increase in appetite caused by problem"(2.1±0.89vs.-1.5±0.90; P=0.04) and "Internal turbulent "(1.2±0.60 vs. 2.2±0.90; p=0.000) in experimental and "consult with her friends" (0.29±0.88 vs. 0.89±0.68; p=0.04) in control group were significant. There was not any significant difference in coping style between the two groups before and after intervention (Table 4).

There were no significant differences in using coping styles within each group before and after intervention except using cognitive-oriented coping style in experimental group (p=0.01; Table 5).

**Table 5:** Differences in coping style within groups before and after intervention

**Intervention** **Group**	**Coping** **Strategies**	**Before**	**After**	**State**	
		Mean(SD)	Mean (SD)	T	P٭
**Control**	Avoidant oriented	07.61±0.60	009.16±2.93	1.97	0.065
	Cognitive oriented	10.22±3.49	11.27±3.5	±1.52	0.14
	Behavioral oriented	09.55±3.43	009.83±2.77	0.33	0.74
**Experimental**	Avoidant oriented	8.47±1.97	8.88±3.60	±0.45	0.65
	Cognitive oriented	10.88±3.60	13.05±4.29	±2.71	0.01
	Behavioral oriented	10.47±2.42	11.70±3.45	±1.35	0.19

٭(p<0.05)

## Discussion

The current results demonstrated that there was a significant difference in hardiness between the two groups after the intervention. It seems that the psychological interventions were effective and could increase the hardiness of participants. In other words, when participants have high level of hardiness they could tolerate the stress of life. 

Previous studies have indicated that individuals with high hardiness would pay more attention to centralized approaches to problem and individuals with low hardiness have more attitudes toward applying centralized approaches to emotions in confronting stress resulting from infertility. Moreover, there are significant relationship between hardiness and social support (11).

Since the role of social supportive networks in our society is weak, this social factor has an important role in promoting resiliency and psychological hardiness in infertile women. Another problem is lack of self-help support groups in our country. Failure to consider the important role of psychologists and/or psychiatrists in infertility clinics is another problem that could contribute to these issues.

Another finding is that there was no significant difference between coping strategies in the two groups of research. Despite other studies, CBT applied here just increased the level of hardiness traits of participants and could not change their coping strategies. Also results indicate that there was significant difference regarding using cognitive-oriented coping style in treatment group before and after intervention. This means that group therapy increases cognitive-oriented approach that may be induced by group therapy and meaningful response to stressful events.

Coping styles that fall under the avoidance-oriented domain tend to be viewed as maladaptive because of their association with greater stress and anxiety (14), physical complains (14,15,24), and mental health problems (17,18,25). In contrast, cognitive-oriented coping styles tend to be viewed as adaptive because of their relationship with less stress and better physical and mental health. For instance, approach-oriented coping styles have been associated with less global stress, and beneficially related to physical health indicators such as illness time loss, as well as indicators of mental health such as anxiety, depression, and psychological distress (14,15,18,19).

The present study emphasizes positive effects of CBT to increase cognitive-oriented coping style in dealing with problems in experimental group after intervention. 

Research findings demonstrated that psychiatric interventions (behavioral, cognitive, psychotherapy) increases the pregnancy chance (26,27); other studies have not found improved pregnancy rates, but have found decreased rates of depression and anxiety (12, 28).

Psychological interventions represent an attractive treatment option, in particular, for infertile patients who are not receiving medical treatment (29).

 Stress management is an effective treatment and this should be offered to patients before, during and after undergoing additional stress of assisted conception treatments (30).

In the resolution or decreasing of depression and anxiety in infertile women, both psychotherapy and CBT are well-established treatments for depression and anxiety (31).

To determine whether a cognitive-behavioral group treatment could lead to a decrease of psychological distress in couples waiting for assisted reproduction, Tarabusi et al. showed that CBT avoids such a 'waiting stress' and could be useful for stimulating discussion and awareness inside the couple (28). Several studies have demonstrated the importance of the mind-body connection and fertility (32).

Domar et al., and Terzioglu, reported that psychiatric interventions led to significant decreases in anxiety and depression and increases in the chance of pregnancy (30, 31). But some studies have shown that various psychological treatments can often contribute to reducing stress but they do rarely increase the possibility of pregnancy (33).

Some reports emphasize on the effect of psychiatric approach on pregnancy rate but some of them emphasize on infertile woman’s mental health and the effect of these approach on psychological distress (8).

Kupka et al. reported that psychological intervention in 14% of cases is conducive to spontaneous pregnancy and that it can be a consequence of reduced stress (27).

Although some studies have noted the first clinical impressions about the usefulness of the body–mind group program in fertility clinics, further research is needed to assess its effectiveness (26,32,34). The support groups can reassure you that you are not alone, that other people do understand you, and provide a place where people "get" what you are going through. An exclusive psychological/psychodynamic point of view on the complexity of infertility is as inadequate as a strictly somatic point of view. Infertility should always be treated as a psychosomatic entirety

## Conclusion

The findings of the present study confirm that psychiatric intervention and counseling have an important role in the mental health of infertile women by development of psychological hardiness and cognitive-oriented coping style.

## Authors’ Contributions

LM reviewed the scientific literature, involved in acquisition of clinical data, conceived and designed the evaluation, interpreted them and helped to draft the manuscript. AKK participated in the evaluation of the statistical analysis, and revised the manuscript. SJ participated in sampling, fallow up stage of research and coordination with participant. All authors read and approved the final manuscript.

## Notes

**Declaration of Interest: **None.

**Clinical trial registration: **IRCT 138903063996N2
